# Transcatheter Mitral Valve Replacement: An Alternate Treatment Methodology for Patients at High Surgical Risk

**DOI:** 10.7759/cureus.2504

**Published:** 2018-04-18

**Authors:** Shafaq Jawed, Ujala Zubair, Zarafshan Zubair

**Affiliations:** 1 MBBS, Jinnah Sindh Medical University (SMC); 2 Medicine, Dow University of Health Sciences (DUHS), Karachi, Pakistan; 3 MBBS, Dow University of Health Sciences (DUHS), Karachi, Pakistan

**Keywords:** mitral valve replacement and repair, transcatheter mitral valve replacement and repair, transcatheter mitral valve replacement and repair, transcatheter mitral valve replacement and repair

## Abstract

Mitral valve regurgitation (MVR) is the reverse passage of blood through the mitral valve when the valve fails to close during ventricle contraction. Transcatheter mitral valve repair and replacement (TMVR) is a recent technology that can benefit MVR patients as an alternative, minimally invasive, and safer solution than conventional open-heart surgical approaches. TMVR uses a catheter guided by ultrasonography to replace or repair the defective valve and has provided better clinical outcomes, decreased hospital stay, and reduced mortality rates. Therefore, TVMR should be made possible worldwide, especially in South-Asian regions where heart conditions such as atrial fibrillation and stroke are major contributing factors in a high postoperative mortality rate.

## Editorial

In developing countries, mitral valve replacement (MVR) usually presents as a complication of myocardial infarction (MI) with an incidence of 13%. The gold standard for the treatment of MVR is surgical MVR (SMVR). In patients > 75 years, SMVR is associated with perioperative complications of a stroke, atrial fibrillation, and low blood volume. The estimated risk of death in SMVR after MI increases by 18% for each increase in age by one year, and the risk of death is 23% with emergency surgery according to a study conducted in Pakistan [1].

Transcatheter mitral valve replacement/repair (TMVR) is a procedure guided by echocardiography and takes three to four hours to complete. Three-dimensional (3D) printing technology allows for patient-specific mitral valve creation which can be implanted via the minimally invasive approach. The MitraClip® (Evalve Inc., Menlo Park, California) was tested on 141 patients with degenerative mitral valve regurgitation and were at prohibitive surgical risk [2]. Slightly improved clinical values were achieved with a 30-day mortality rate of 6.3% and a one-year mortality rate of 23.6%. Additionally, TVMR procedures resulted in shortened hospital stays and decreased the frequency of re-hospitalization [3]. A study comparing TMVR with SMVR techniques yielded significant results in favor of TMVR therapy for reduced hospital stay (12.6 ± 24.7 days for TMVR vs 22.8 ± 18.4 days for SMVR). Moreover, TMVR has a reduced rate of postoperative complications in comparison to SMVR such as cardiac arrest (6.8% vs 6.9%), stroke (0% vs 8.5%), and reduced in-hospital mortality rates (1.3% vs 13.8%) [4].

Giamo et al. report his study results which were conducted among 225 patients. These patients underwent cardiac resynchronization therapy (CRT). A total of 77 patients failed to respond to CRT. Of these patients, 30 underwent MitraClip® repair. These patients had New York Heart Association (NHYA) class III or IV heart failure with moderate to severe or severe functional mitral regurgitation (FMR). Periprocedural death occurred in one patient. Patients were followed at one, three, six, 12, 18, and 24 months; 17% patients died at 24 months follow up. Among the surviving patients, there was a significant improvement in NHYA heart failure class and FMR at six and 12 months (p<0.001, p<0.001 respectively). However, no improvement was recorded at 12 to 24 months interval among survivors [4]. The four-year outcome from the EVEREST II trial showed no difference in mortality among patients treated with percutaneous approach and surgery among patients with grade 3+ or 4+ Mitral regurgitation [5].

TMVR requires expertise for computed tomography (CT) analyses of preoperative measures such as mitral valve size. TMVR accounts for a significant reduction in postoperative complication rates and reduced in-hospital stay as compared to open heart surgery. Pakistan reports a high number of mitral valve regurgitation. Due to poor post-operative care, TMVR would provide a safe surgical option for the treatment of mitral valve regurgitation.
